# Fault Diagnosis in Wind Turbine Current Sensors: Detecting Single and Multiple Faults with the Extended Kalman Filter Bank Approach

**DOI:** 10.3390/s24030728

**Published:** 2024-01-23

**Authors:** Mohammed Abbas, Houcine Chafouk, Sid Ahmed El Mehdi Ardjoun

**Affiliations:** 1 IRSEEM/ESIGELEC Laboratory, Normandy University of Rouen, 76000 Rouen, France; mohammed.abbas@univ-sba.dz; 2 IRECOM Laboratory, Djillali Liabes University, Sidi Bel-Abbes 22000, Algeria

**Keywords:** diagnostic, DFIG, wind turbine, extended Kalman filter, current fault sensor

## Abstract

Currently, in modern wind farms, the doubly fed induction generator (DFIG) is commonly adopted for its ability to operate at variable wind speeds. Generally, this type of wind turbine is controlled by using two converters, one on the rotor side (RSC) and the other one on the grid side (GSC). However, the control of these two converters depends mainly on current sensors measurements. Nevertheless, in the case of sensor failure, control stability may be compromised, leading to serious malfunctions in the wind turbine system. Therefore, in this article, we will present an innovative diagnostic approach to detect, locate, and isolate the single and/or multiple real-phase current sensors in both converters. The suggested approach uses an extended Kalman filter (EKF) bank structured according to a generalized observer scheme (GOS) and relies on a nonlinear model for the RSC and a linear model for the GSC. The EKF estimates the currents in the converters, which are then compared to sensor measurements to generate residuals. These residuals are then processed in the localization, isolation, and decision blocks to precisely identify faulty sensors. The obtained results confirm the effectiveness of this approach to identify faulty sensors in the abc phases. It also demonstrates its ability to overcome the nonlinearity induced by wind fluctuations, as well as resolves the coupling issue between currents in the fault period.

## 1. Introduction

Driven by economic expansion, global energy demand has experienced exceptional growth in recent years. However, this expansion raises major concerns due to the ecological issues it entails, as some countries rely heavily on thermal power plants. The use of renewable energy sources has, therefore, become inevitable to mitigate these effects [1,2,3,4]. Among the envisaged solutions, wind systems emerge as one of the most promising options [5,6,7]. Their versatility is evident in various contexts, ranging from grid-connected farms or isolated sites to configurations of hybrid energy systems [8,9].

It should be noted that the majority (70%) of wind turbines typically rely on a doubly fed induction generator (DFIG) [9,10,11,12]. This machine has a stator winding connected to the grid through a transformer, while the rotor is connected to the electrical network through AC/DC/AC converters. Thanks to this architecture, the wind turbine can operate in a variable speed range, varying between ±30% around the synchronous speed. This characteristic helps reduce the size of the converters, offering a significant economic advantage over other types of generators [13].

These converters play a central role in controlling the power exchanged with the grid. These operations are carried out using specific control techniques applied to these converters, which constitute key elements of the underlying technology. The entire set of control techniques relies primarily on current measurements [14]. These sensors are prone to numerous faults that impair measurement quality. Based on industrial and field statistics [15,16,17], current sensor faults are classified as significant issues arising from high power density and electromagnetic interference [18].

In the case of current sensor failure, the stability of the current control behavior is compromised, leading to a total or partial loss of converter control. These failures can lead to serious malfunctions in the wind power system, currently justifying its shutdown and disconnection from the grid [19]. These faults lead to high maintenance costs associated with the replacement of faulty components, and significant energy losses [14].

In order to ensure the service continuity of the wind power system and anticipate any degradation, it is imperative to establish an effective diagnostic method enabling the detection, localization, and isolation of faults in the current sensors. These diagnostic methods are generally categorized into three types: signal processing, model-based approaches, and methods using artificial intelligence [20,21,22].

Concerning the model-based approach, several researchers have chosen to use it to detect and isolate faults, especially those related to current sensors. These approaches utilize observer theory, such as unknown input observers based on the DFIG model with fuzzy logic [23], or sliding mode observers based on a new reach law [24]. Additionally, the extended Kalman filter (EKF) is used for fault detection and isolation (FDI), and the reconfiguration of the system in the case of current sensor faults [25]. Another method, which relies on a bank of Luenberger observers to diagnose faults in DFIG sensors, is proposed in [26]. Other diagnostic techniques for these faults are being considered, such as the use of the Takagi-Sugeno (TS) model based on the state model of the DFIG, structured according to the multiple observers set [27], or according to the Luenberger observer set [28]. Another method, presented in [29], is based on the use of the TS model with Lyapunov theory to diagnose these faults.

Furthermore, other diagnostic approaches are developed to detect and locate multiple and simultaneous faults for the current sensors of the DFIG. This is done through a bank of observers based on a Kalman filter, structured according to the generalized observer scheme (GOS), as described in [30,31], or structured according to the dedicated observer scheme (DOS), as presented in [32,33].

However, the majority of the proposed diagnostic methods depend on the DFIG state model in the αβ or dq reference frames. Although faults have been diagnosed on this reference, it should be pointed out that no sensors are available to measure the currents of these references. Furthermore, creating faults at these references in real time is not feasible, as they are fictitious mathematical representations designed to simplify DFIG modeling with the aim of controlling it. It should be noted that the currents of references alpha and beta encompass all the currents of phases a, b, and c. For example, the $I_{\beta }$ current is associated with the *Ic* and *Ib* currents. Thus, in the case of a fault in the $I_{\beta }$ current, it is possible that the sensor of phase (b), phase (c), or both, is responsible for the fault [34].

However, these methods prove to be insufficient to identify accurately the true sensor faults, when they occur in a single phase (a), (b), or (c), or simultaneously in two sensors in the phases (ab), (ac), or (bc). The majority of the above-mentioned publications have tackled the problem of nonlinearity by simplifying the DFIG model in the form of a linear variable parameter (LVP) system based on the variation in mechanical speed.

However, this model is less accurate due to the variation in this parameter. Other studies have also adopted the TS model, which suffers from the problem of chattering [35]. Thus, both models can lead to inaccurate current predictions, resulting in inaccurate diagnosis. In addition, previous studies have not addressed possible faults in the GSC sensors, which are essential for managing energy exchanges between the generator and the grid.

Thus, this article proposes two parallel diagnostic approaches, both based on the EKF structured according to the GOS, to detect, isolate, and localize single and multiple simultaneous faults in converter current sensors (RSC and GSC). The contributions of this paper can be summarized in the following points:

- The proposed approach is capable of identifying current sensor faults in both converters in the three-phase reference frame (a, b, c).
- The diagnostic algorithm is based on the estimation and prediction of currents using a nonlinear and linear Kalman filter with a logic combination block.
- The nonlinearity problem in our system is solved by Jacobi’s discrete-time method.
- The developed fault diagnosis technique is applied for RSC and GSC current sensors based on the EKF.

The article is structured as follows: Section 2 presents the mathematical model of the DFIG. Section 3 introduces the sensor fault diagnostic approach. The results obtained and discussions are included in Section 4. Finally, the conclusion and perspectives of this article are presented in Section 5.

## 2. Wind Power System Modelling

**Figure 1:**
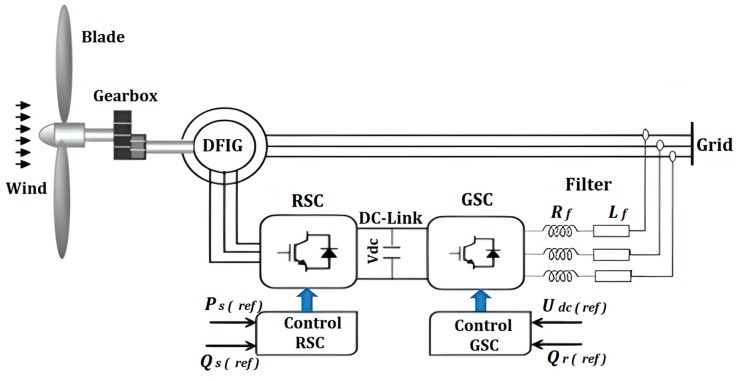
Wind power system based on a DFIG.

The architecture of our wind power system, based on a DFIG with two power converters, is shown in Figure 1.

### 2.1. DFIG Modeling

The model of the DFIG is modeled in the park transformation reference frame *dq* linked to the rotating magnetic field, in order to control and detect its faults.

The voltage equations of the DFIG in the *dq* reference frame are [36]:

(1) $$\begin{array}{c} V_{ds}=R_{s}I_{ds}+\frac{{d\varphi }_{ds}}{dt}-\omega_{s}\varphi_{qs}\\ V_{qs}=R_{s}I_{qs}+\frac{{d\varphi }_{qs}}{dt}+\omega_{s}\varphi_{ds}\\ V_{dr}=R_{r}I_{dr}+\frac{{d\varphi }_{dr}}{dt}-\omega_{r}\varphi_{qr}\\ V_{qr}=R_{r}I_{qr}+\frac{{d\varphi }_{qr}}{dt}+\omega_{r}\varphi_{dr} \end{array}$$

The mechanical equation of the DFIG is expressed as:

(2) $$J\frac{d\Omega }{dt}={T_{em}-T}_{r}+T_{f}$$

The electromagnetic torque is expressed as:

(3) $$T_{em}=p\frac{M}{L_{s}}\left(I_{dr}\varphi_{qs}-I_{qr}\varphi_{ds}\right)$$

where:

$V_{ds}$, $V_{qs}$ and $I_{ds},{\;I}_{qs}$ are the voltages [V] and currents [A] of stator phases *d* and *q* in the *dq* frame, respectively.${\;V}_{dr}$, $V_{qr}{\;and\;I}_{dr},I_{qr}$ are the voltages [V] and currents [A] of rotor phases *d* and *q* in the *dq* frame, respectively. $\varphi_{ds},\;\varphi_{qs},\varphi_{dr},\varphi_{qr}$ are the stator and rotor fluxes [Wb], respectively, in frame of reference dq. $R_{s},R_{r}$ are stator resistance and rotor resistance, respectively. $L_{s}$ is stator inductances [H]. M is mutual inductances [H]. $\omega_{s}$ and $\omega_{r}$ are the stator and rotor pulsations [rad/s], respectively. J is the inertia of the DFIG $\left[K\cdot m^{2}\right]$; p is the number of pairs of poles.

$T_{em},T_{r},T_{f}$ are the generator and resistive and frictional torque [N·m], respectively.

To reduce the computations and avoid the transition between the abc and dq reference frame, we will model the state of the DFIG in the reference frame (α, β), which is related to the rotating magnetic field. It is expressed as follows:

(4) $$\frac{dx_{1}}{dt}=A_{1}x_{1}+B_{1}U_{1}$$

(5) $$y_{1}=C_{1}x_{1}$$

(6) $$x_{1}={\left[I_{\alpha s}{,I}_{\beta s}{,I}_{\alpha r}{,I}_{\beta r}\right]}^{T},U_{1}={\left[V_{\alpha s},V_{\beta s},V_{\alpha r},V_{\beta r}\right]}^{T}$$

where $x_{1}$ is the state vector of the stator and rotor current expressed in the αβ reference frame. $U_{1}$ is the control input of the stator and rotor voltage in the αβ reference frame, and $y_{1}$ is the output vector.

The state matrix $A_{1}$ is:

(7) $$A_{1}=\left[\begin{array}{cccc} -\frac{R_{s}}{\sigma L_{s}} & \frac{{\omega M}^{2}}{\sigma L_{s}L_{r}} & \frac{{MR}_{r}}{\sigma L_{s}L_{r}} & \frac{M\omega }{\sigma L_{s}}\\ -\frac{{\omega M}^{2}}{\sigma L_{s}L_{r}} & -\frac{R_{s}}{\sigma L_{s}} & -\frac{M\omega }{\sigma L_{s}} & \frac{{MR}_{r}}{\sigma L_{s}L_{r}}\\ \frac{R_{s}M}{\sigma L_{s}L_{r}} & -\frac{M\omega }{\sigma L_{r}} & -\frac{R_{r}}{\sigma L_{r}} & -\frac{\omega }{\sigma }\\ \frac{M\omega }{\sigma L_{r}} & \frac{R_{s}M}{\sigma L_{s}L_{r}} & -\frac{\omega }{\sigma } & -\frac{R_{r}}{\sigma L_{r}} \end{array}\right]$$

The input matrix $B_{1}$ is:

(8) $$B_{1}=\left[\begin{array}{cccc} \frac{1}{\sigma L_{s}} & 0 & -\frac{M}{\sigma L_{s}L_{r}} & 0\\ 0 & \frac{1}{\sigma L_{s}} & 0 & -\frac{M}{\sigma L_{s}L_{r}}\\ -\frac{M}{\sigma L_{s}L_{r}} & 0 & \frac{1}{\sigma L_{r}} & 0\\ 0 & -\frac{M}{\sigma L_{s}L_{r}} & 0 & \frac{1}{\sigma L_{r}} \end{array}\right]$$

where $\sigma =\frac{M^{2}}{L_{s}L_{r}}\;$ is the leakage coefficient, $L_{r}$ is the cyclic rotor inductances [H], and ω is the angular frequency of the rotor [rad/s].

The output matrix $C_{1}\;$ is:

(9) $$C_{1}=\left[\begin{array}{cccc} 1 & 0 & 0 & 0\\ 0 & 1 & 0 & 0\\ 0 & 0 & 1 & 0\\ 0 & 0 & 0 & 1 \end{array}\right]$$

### 2.2. Modeling of the GSC Connection

This model consists of the GSC, RL filter, and the grid. The modeling of the GSC connection in the αβ reference frame is represented as follows [37]:

(10) $$V_{\alpha f}=R_{f}I_{\alpha g}+\frac{{dI}_{\alpha g}}{dt}+V_{\alpha g}$$

(11) $$V_{\beta f}=R_{f}I_{\beta g}+\frac{{dI}_{\beta g}}{dt}+V_{\beta g}$$

where $R_{f}$ is the filter resistive.

The state model of GSC, RL filter, and the grid is represented as follows:

(12) $$\frac{dx_{2}}{dt}=A_{2}x_{2}+B_{2}U_{2}$$

(13) $$y_{2}=C_{2}x_{2}$$

where:

(14) $$x_{2}={\left[I_{\alpha g}{,I}_{\beta g}{,R}_{f}\right]}^{T}$$

(15) $$U_{2}={\left[V_{\alpha f},V_{\beta f},V_{\alpha g},V_{\beta g}\right]}^{T}$$

where $x_{2}$ is the state vector formed of the two currents of the converter expressed in the αβ reference frame and $U_{2}$ is the input vector composed of the GSC and grid voltages in the αβ reference frame noted, respectively, $V_{\alpha f},V_{\beta f}$, $V_{\alpha g},V_{\beta g}$, and $y_{2}$ is the output vector.

The state matrix $A_{2}$ is:

(16) $$A_{2}=\left[\begin{array}{ccc} -\frac{R_{f}}{L_{f}} & 0 & 0\\ 0 & -\frac{R_{f}}{L_{f}} & 0\\ 0 & 0 & 1 \end{array}\right]$$

The input matrix $B_{2}$ is:

(17) $$B_{2}=\left[\begin{array}{cccc} \frac{1}{L_{f}} & 0 & \frac{-1}{L_{f}} & 0\\ 0 & \frac{1}{L_{f}} & 0 & -\frac{1}{L_{f}}\\ 0 & 0 & 0 & 0 \end{array}\right]$$

The output matrix $C_{2}$ is:

(18) $$C_{2}=\left[\begin{array}{ccc} 1 & 0 & 0\\ 0 & 1 & 0\\ 0 & 0 & 1 \end{array}\right]$$

## 3. The Sensor Fault Diagnosis Approach

**Figure 2:**
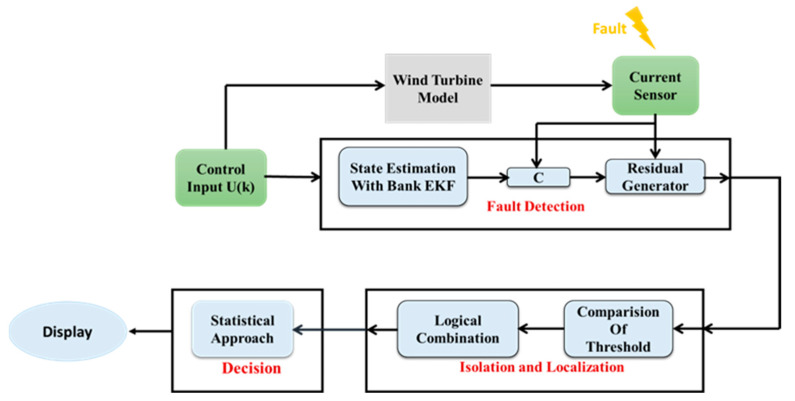
Current sensor diagnostic unit.

In our article, we have presented a model-based diagnostic approach. The latter relies on the use of the EKF to diagnose current sensors. The approach developed is divided into two identical diagnostic units: the first one for the current sensors on the RSC and the second one for the current sensors on the GSC. Each unit is structured as shown in Figure 2, based on the discrepancy (residue) between the sensor measurements and the signals estimated by the Kalman filter bank from the state model. This allows the generating of residues used as fault indicators in the first detection step. The residues are then processed in a phase of localization and isolation to identify the faulty sensors in each phase. Finally, the residues undergo a decision phase aiming to differentiate between faulty sensors and measurement disturbances, in order to avoid false alarms.

The diagnostic approach can be divided into three steps for each unit, as follows.

### 3.1. Fault Detection

This step relies on the EKF (a method for estimating and predicting unmeasurable or highly noisy states in discrete time). This technique relies on the measured inputs/outputs of the system and on the discrete state space system model [38], defined as follows:

(19) $$\begin{array}{ll} x_{k+1} & ={f(x}_{k}{,U}_{k})+W_{k}\\ & =A_{d}x_{k}+B_{d}U_{k}+W_{k} \end{array}$$

(20) $$\begin{array}{ll} y_{k} & ={h(x}_{k})+V_{k}\\ & ={C_{d}x}_{k}+V_{k} \end{array}$$

where:

(21) $$A_{d}=e^{{AT}_{s}}=I+{AT}_{s}$$

(22) $${\;B}_{d}=B{\;T}_{s}$$

(23) $${C_{d}=CT}_{s}$$

where:

$W_{K}$ and $V_{K}$ are Gaussian noises, respectively, of the process and measurement. $\;T_{s}$ is time sampling.

And the Kalman filter application for the RSC and GSC models are defined as follows.

#### 3.1.1. EKF Application on the RSC

**Figure 3:**
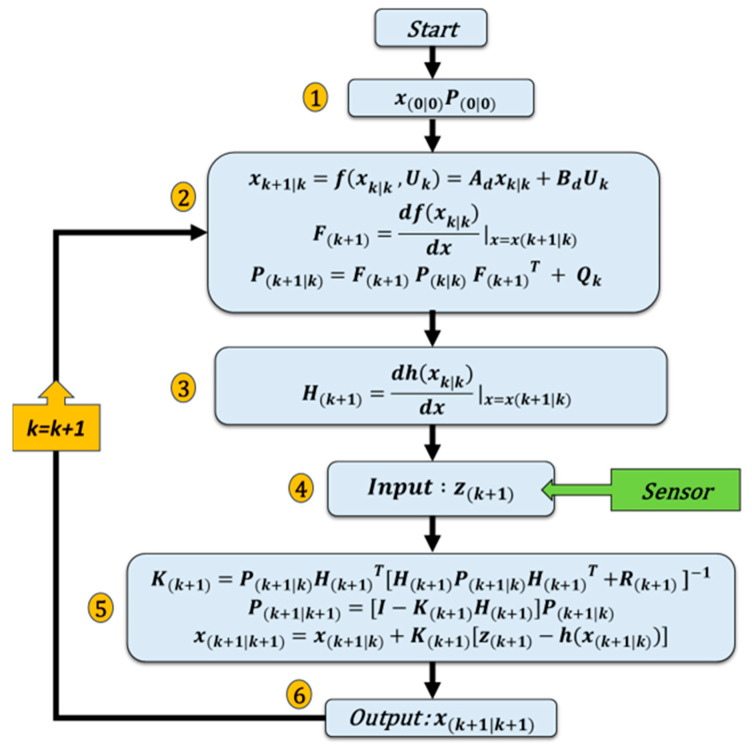
The flowchart of the EFK algorithm.

The DFIG model is nonlinear due to the dependence of the mechanic velocity on the wind speed. In order to solve this nonlinearity problem, the EKF is designed, integrating the calculation of the Jacobian partial derivative [39]. This method approximates the nonlinear system to a linear system around the operating points, thus facilitating the estimation of rotor and stator currents. Figure 3 illustrates the algorithm for applying the EKF to the DFIG model. Here are the different steps presented in Figure 3:

1. Initialization step: calculates the initial state vector at time k = 0 and the covariance matrix associated with the initially estimated state.
2. The prediction phase: calculates the system state, the Jacobian of the nonlinear matrix F with respect to the state variables x and the covariance matrix.
3. Calculate the Jacobian of the nonlinear matrix H with respect to the state variables x.
4. Acquisition of a new current measurement.
5. Update phase: Kalman gain calculation, estimate update, covariance matrix update.
6. Estimated state variables used.

where:

${f(x}_{k}{,U}_{k})$ is the state transition model, ${h(x}_{k})$ is the measurement model, $F_{\left(k+1\right)}$ is the Jacobian matrix for the state transition, $H_{\left(k+1\right)}$ is the Jacobian matrix for the state transition; $P_{\left(k|k\right)}$ is the error covariance; $K_{\left(k+1\right)}$ is Kalman gain, $Q_{k}$ is the process noise covariance matrix and, $R_{k}$ is the measurement noise covariance matrix.

#### 3.1.2. EKF Application on the GSC

The model on the network side is linear, as the angular velocity of our system is imposed by the network and fixed at ω_s_ = 2 π 50 rad/s. The application of the EKF to this model follows the previous algorithm but without the linearization step, and without the calculation of matrices F and H.

**Figure 4:**
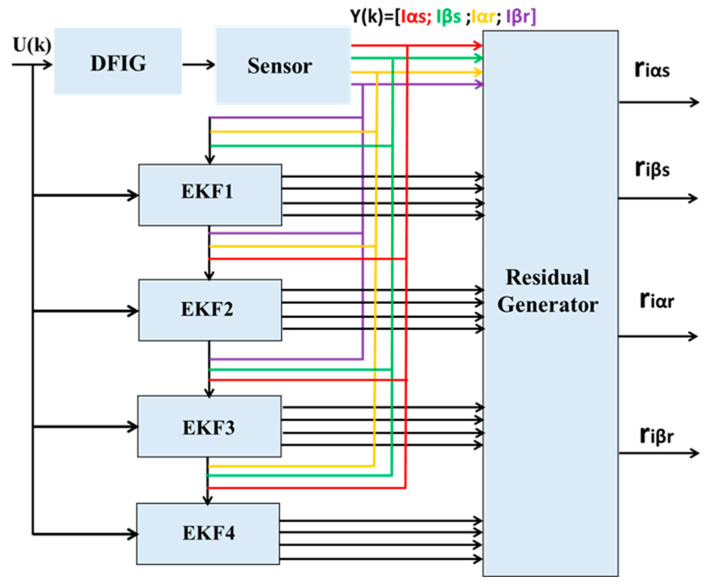
Structure of the Kalman GOS bank on the RSC.

In order to detect faults in multiple current sensors on the RSC and GSC in the αβ reference frame, two banks of EKF, structured according to the GOS, are implemented. The first bank consists of four Kalman observers for faults in the stator and rotor current sensors on the RSC, as illustrated in Figure 4.

**Figure 5:**
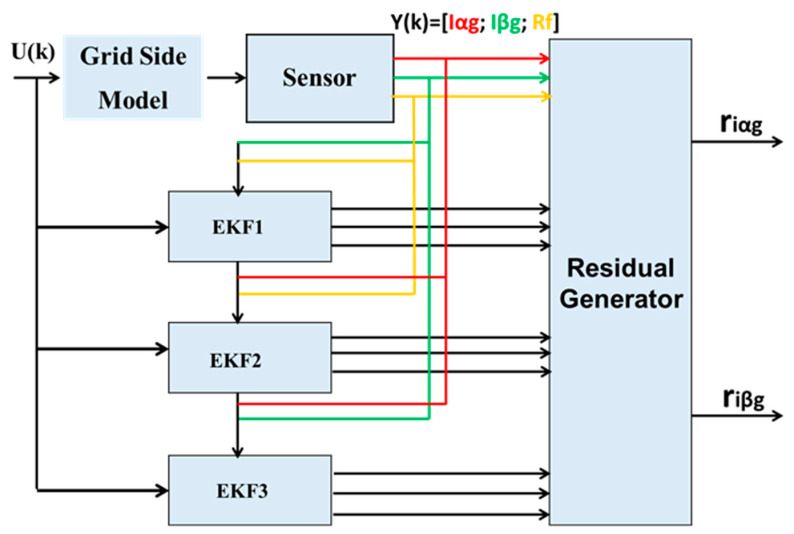
Structure of the Kalman GOS bank on the GSC.

The second bank consists of three Kalman observers, two for current sensor faults and one for the resistance of the grid-side filter, as shown in Figure 5. Thus, the number of observers corresponds to the number of sensors, where the nth observer is driven by all inputs U and outputs Y of the system, except for the nth output. Each Kalman filter is designed as an observer under normal conditions, and is sensitive to a particular fault, producing an output estimate ŷ.

The difference between the measured signals and the signals estimated by each EKF represents the residual values used to detect faults, which are calculated as follows:

(24) $$\left\{\begin{array}{c} r_{i\alpha s}={I^{\prime }}_{s\alpha }-I_{s\alpha }\\ r_{i\beta s}={I^{\prime }}_{s\beta }-I_{s\beta }\\ r_{i\alpha r}={I^{\prime }}_{r\alpha }-I_{r\alpha }\\ r_{i\beta r}={I^{\prime }}_{r\beta }-i_{r\beta } \end{array}\right.$$

(25) $$\left\{\begin{array}{c} r_{i\alpha g}={I^{\prime }}_{\alpha g}-I_{\alpha g}\\ r_{i\beta g}={I^{\prime }}_{\beta g}-I_{i\beta g} \end{array}\right.$$

### 3.2. Localization and Isolation

Our aim is to locate and isolate faults in the real abc frame (phase a, b, or c) for the RSC and GSC. It should be noted that fault location in the αβ frame is complex due to the coupling between the phases of the currents. For example, a fault affecting the current sensor in phase b or c will influence both components in the αβ reference frame of the current, resulting in changes in the residuals $r_{\alpha }\;$ et $r_{\beta }$.

To solve this problem, we have used the Clarke transformation [40] to convert errors from the αβ reference frame to errors in the abc reference frame. A logical combination is then used for both models, ensuring accurate fault detection in the sensors. This logical combination is calculated as follows:

(26) $$\left[\begin{array}{c} r_{iar}\\ r_{ibr}\\ r_{icr} \end{array}\right]=\left[\begin{array}{cc} 1 & 0\\ -\frac{1}{2} & \frac{\sqrt{3}}{2}\\ -\frac{1}{2} & -\frac{\sqrt{3}}{2} \end{array}\right]\left[\begin{array}{c} r_{r\alpha }\\ r_{r\beta } \end{array}\right]$$

(27) $$\left[\begin{array}{c} r_{iag}\\ r_{ibg}\\ r_{icg} \end{array}\right]=\left[\begin{array}{cc} 1 & 0\\ -\frac{1}{2} & \frac{\sqrt{3}}{2}\\ -\frac{1}{2} & -\frac{\sqrt{3}}{2} \end{array}\right]\left[\begin{array}{c} r_{i\alpha g}\\ r_{i\beta g} \end{array}\right]$$

The logical combination x is defined by a flag based on the comparison of each residue with its threshold ${\;S}_{i}$. The two possibilities for the flag of each current sensor are given as follows:

If $\left|r_{i}\right|\geq S_{i}\;$ Presence of a fault, so the flag equals 1.

If $\left|r_{i}\right|<S_{i\;}$ Absence of a fault, so the flag equals 0.

With three sensors on each side and two flag states, we have eight possible cases ($2^{3}=8$).

The logical combination for the eight possible cases for the three current sensors x = [a b c] is defined as follows:

- If $\left|r_{iaj}\right|\geq S_{iaj}$ and $\left|r_{ibj}\right|<S_{ibj}\;and\;\left|r_{icj}\right|<S_{icj\;}$ fault on sensor a; x = [1 0 0].
- If $\left|r_{iaj}\right|<S_{iaj}$ and $\left|r_{ibj}\right|\geq S_{ibj}\;and\;\left|r_{icj}\right|<S_{icj}\;$ fault on sensor b; x = [0 1 0].
- If $\left|r_{iaj}\right|<S_{iaj}$ and $\left|r_{ibj}\right|<S_{ibj}\;and\;\left|r_{icj}\right|\geq S_{icj\;}$ fault on sensor c; x = [0 0 1].
- If $\left|r_{iaj}\right|\geq S_{iaj}$ and $\left|r_{ibj}\right|\geq S_{ibj}\;and\;\left|r_{icj}\right|<S_{icj}$ fault on sensor a and b; x = [1 1 0].
- If $\left|r_{iaj}\right|\geq S_{iaj}$ and $\left|r_{ibj}\right|<S_{ibj}\;and\;\left|r_{icj}\right|\geq S_{icj}$ fault on sensor a and c; x = [1 0 1].
- If $\left|r_{iaj}\right|<S_{iaj}$ and $\left|r_{ibj}\right|\geq S_{ibj}\;and\;\left|r_{icj}\right|\geq S_{icj}$ fault on sensor b and c; x = [0 1 1].
- If $\left|r_{iaj}\right|\geq S_{iaj}$ and $\left|r_{ibj}\right|\geq S_{ibj}\;and\;\left|r_{icj}\right|\geq S_{icj}\;$ fault on sensor a, b and c; x = [1 1 1].
- If $\left|r_{iaj}\right|<S_{iaj}$ and $\left|r_{ibj}\right|<S_{ibj}\;and\;\left|r_{icj}\right|<S_{icj}$ x = [0 0 0].
  with:
  j = r: for the RSC.
  and:
  j = f: for GSC.

### 3.3. Decision

It is essential to distinguish between current sensor faults and measurement noise to avoid false alarms. Additionally, it is necessary to determine the time of occurrence and disappearance of the fault. Statistical tests are employed in this approach to prevent false alarms and to define the fault occurrence duration. The Page–Hinkley (P-H) statistical test has been chosen in our study. This test is based on the principle of detecting an abrupt change in the mean of a signal [41,42].

The detection problem consists of running two tests in parallel. The test is performed between the no-change hypothesis ${(H}_{0}$:r>n) and the hypothesis of change ($H_{1}$: r≤n), where *r* is the time of change, and *n* is the first observation. The application of the P-H test requires the determination of *m* et $\delta_{i}$ which are, respectively, the mean of the residual signal and the standard deviation of this signal.

The procedure involves running two tests in parallel. The first test detects an increase in the average, as follows:

(28) $$U_{n}=\sum_{i=1}^{n}\left(x_{i}-m_{0}-\frac{\delta_{i}}{2}\right),\;n\geq 1,\;U_{0}=0$$

(29) $$m_{n}=\min_{0\leq k\leq n}{(U}_{k}),n\geq 1$$

The default value is produced when $U_{n}-m_{n}\geq \lambda$, with a threshold of λ. In other words, the no change hypothesis $H_{1}$ when $U_{n}$ is greater than λ is used.

The second test allows for detecting a decrease in the average and is calculated by:

(30) $$M_{n}=\max_{0\leq k\leq n}{(U}_{k}),n\geq 1$$

The flaw occurs when $M_{n}-U_{n}\geq \lambda$, with a threshold of λ. In other words, we decide $H_{1}$ when $U_{n}$ is less than λ.

## 4. Test Results

**Figure 6:**
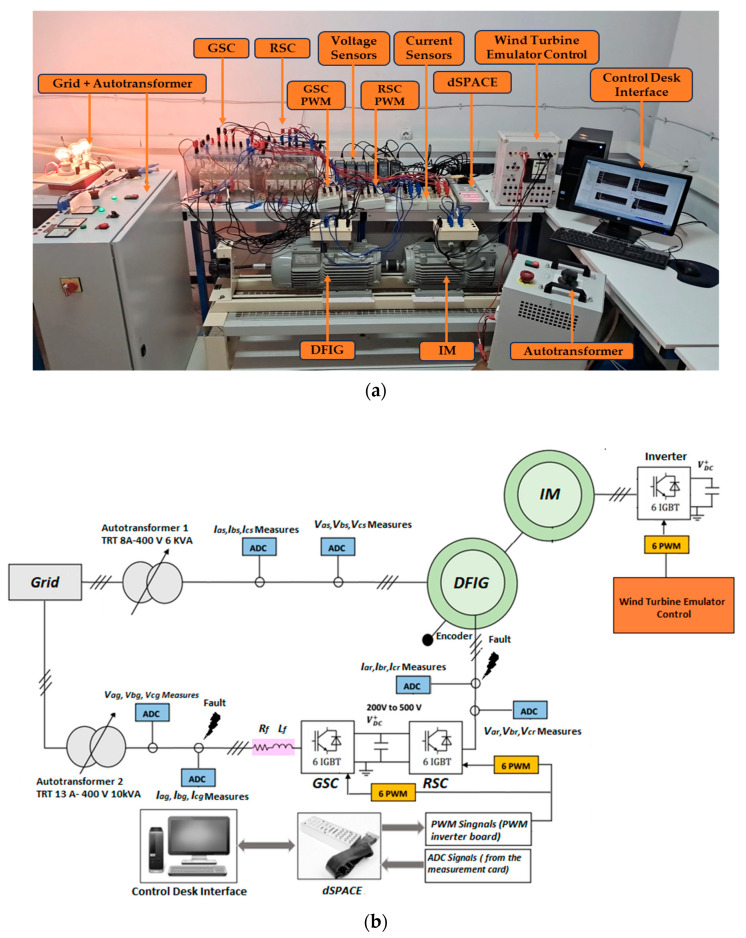
Experimental bench of the wind energy conversion chain emulator: (**a**) photo, (**b**) schematic diagram.

In order to validate the diagnostic approaches for faults in current sensors and assess the performance of detection and localization of faulty current sensors in a DFIG based wind turbine, two tests were conducted. These tests were inspired by the test bench presented in Figure 6.

**Figure 7:**
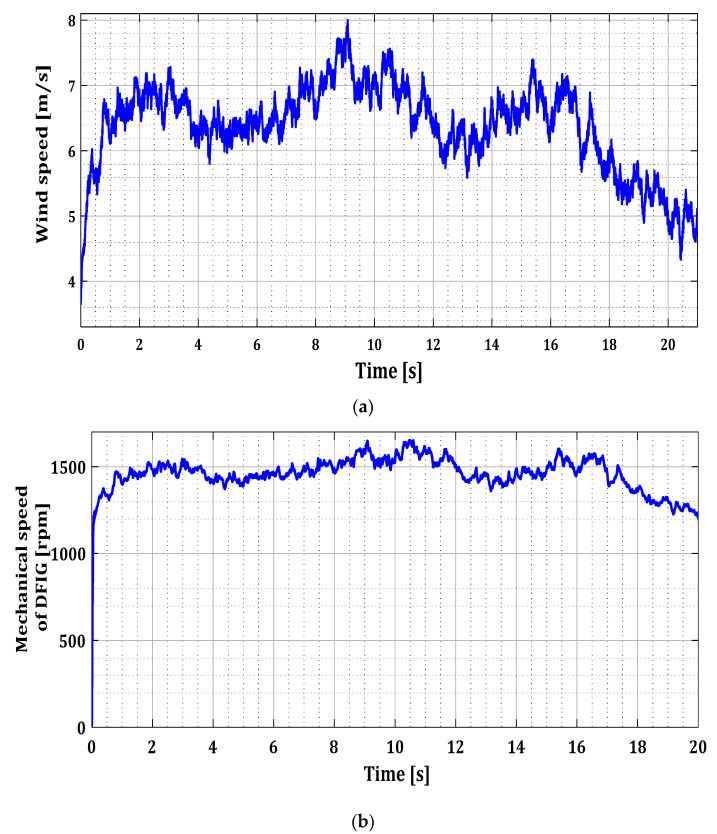
(**a**) Wind speed profile and (**b**) DFIG mechanical speed.

The first test aims to apply a fault to one of the three sensors of phases (a), (b), or (c). And the second test involves applying simultaneous faults to two sensors. In each test, all possible fault scenarios for the current sensors of phases (a), (b), or (c) of each converter (RSC and GSC) were tested. Table 1 indicates the fault period, the faulty sensor, and the type of faults. In both tests we have applied a random wind profile to our system represented by Figure 7.

These tests enable us to evaluate the effectiveness of the proposed approach in detecting and localizing sensor faults, thereby providing quantitative results on the system performance.

### 4.1. Scenario of Unique Defects

**Figure 8:**
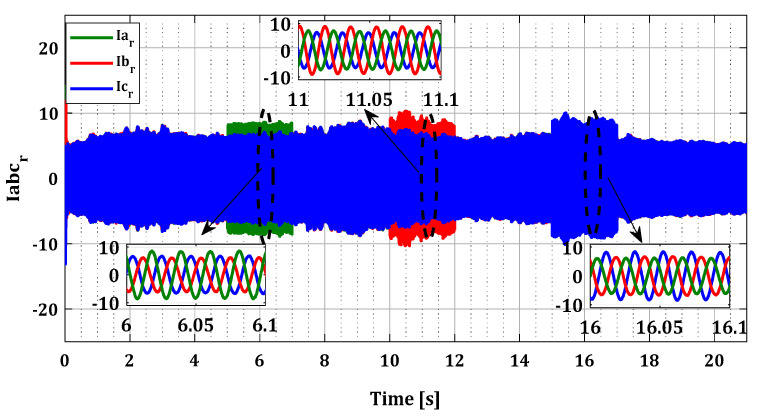
Rotor currents $I_{ar}$, $I_{br}$, and $I_{cr}$ for single faults.

**Figure 9:**
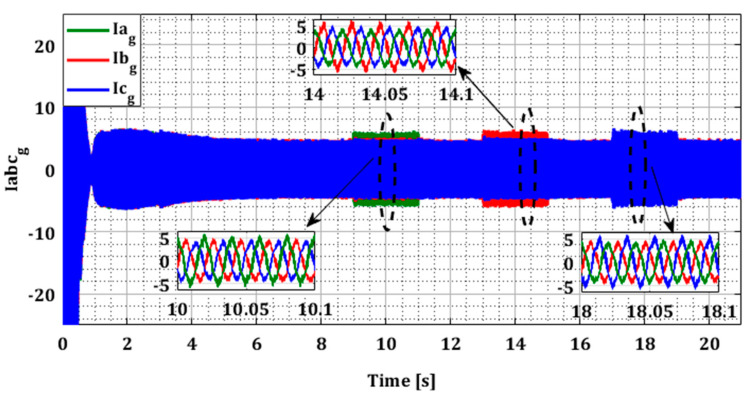
Grid currents $I_{ag}$, $I_{bg}$, and $I_{cg}$ for single faults.

In this test, faults were intentionally introduced on each sensor of phases a, b, and c during successive periods T1, T2, and T3 for both converters, as indicated in Table 1. Figure 8 and Figure 9 illustrate the current profiles for both converters, highlighting the increase in current in each phase during the sensor failure period.

**Figure 10:**
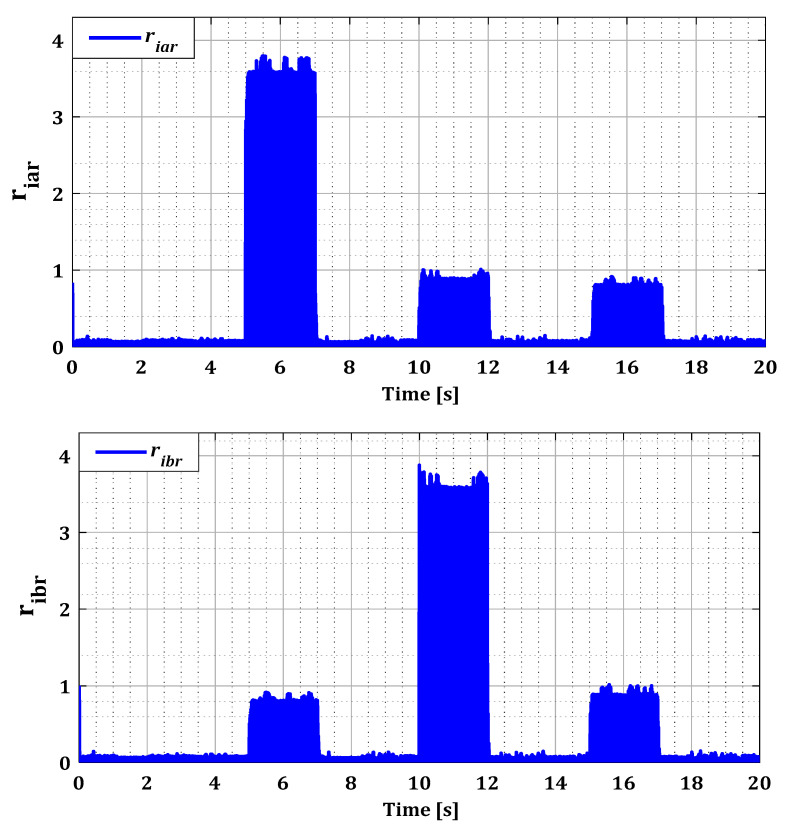

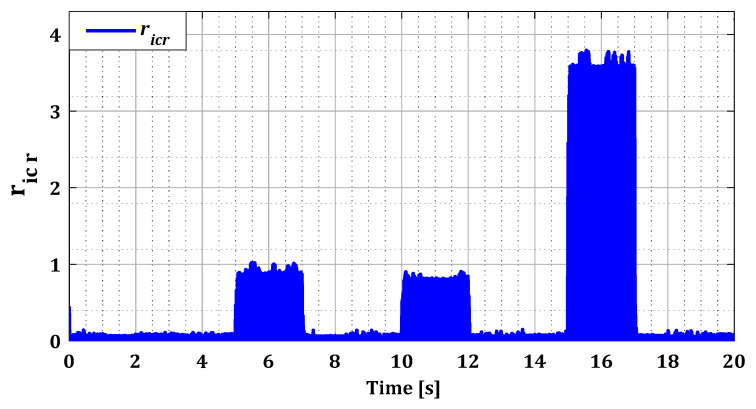
Residues $r_{iar}$, $r_{ibr}$, and $r_{icr}$ for single faults.

**Figure 11:**
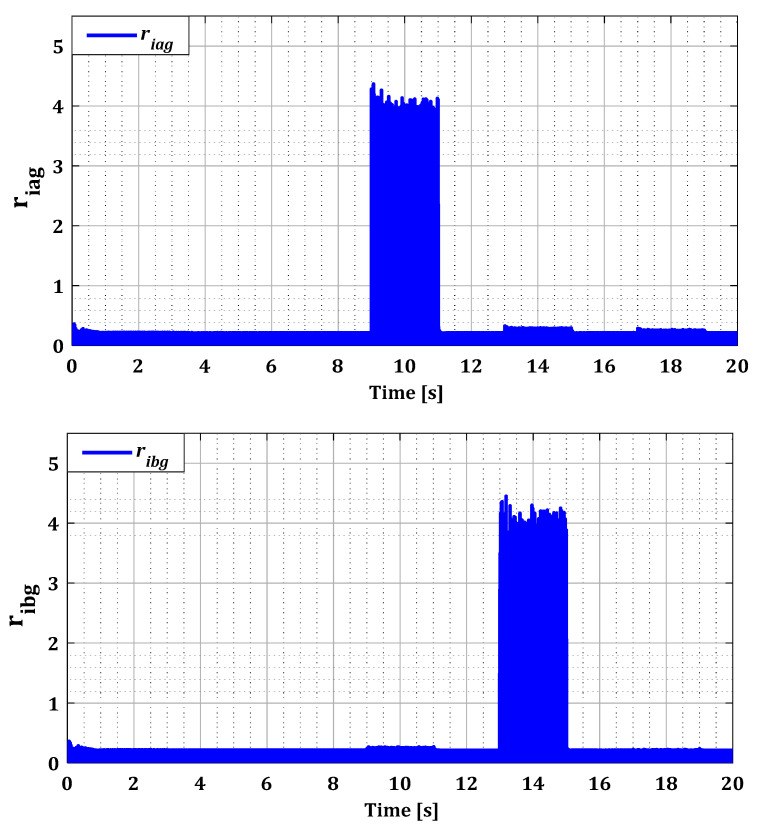

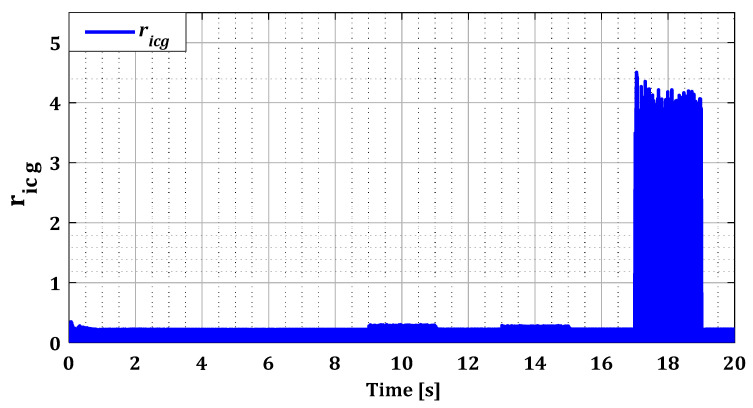
Residues $r_{iag}$, $r_{ibg}$, and $r_{icg}$ for single faults.

To assess the ability of the localization and isolation block to accurately identify these faults, it is essential to examine the residuals generated by this block for both converters, as illustrated in Figure 10 and Figure 11. In the normal operating state of the current sensors, all estimation errors are zero, as demonstrated by Figure 10 and Figure 11. This observation highlights the effectiveness of our approach based on the Kalman filter for accurate estimation of the currents in both converters. Furthermore, this method has satisfactorily addressed the nonlinearity issue of DFIG induced by variations in wind speed, as proven by the zero values of the rotor current estimation residuals in Figure 10.

According to Figure 10 and Figure 11, it was observed that the value of the residuals for each current phase was influenced by the failure of its sensor during fault period for both converters. This proves that the Kalman filter bank detected the existence of the fault, leading to high estimation errors. Then the localization phase identified the location of the fault with its period in the abc reference after the Clark transformation but without accuracy. It is because the residual values of the undamaged current sensors in RSC have also been affected by the fault due to the influence produced by the coupling between the rotor phases. For example, in the case of a sensor fault in phase (a) during period T1, a change is observed in the value of the residuals $r_{ibr}$ and $r_{icr}$, but less pronounced than the value of the residual $r_{iar}$. This effect has been eliminated by the logical combination in the isolation phase.

**Figure 12:**
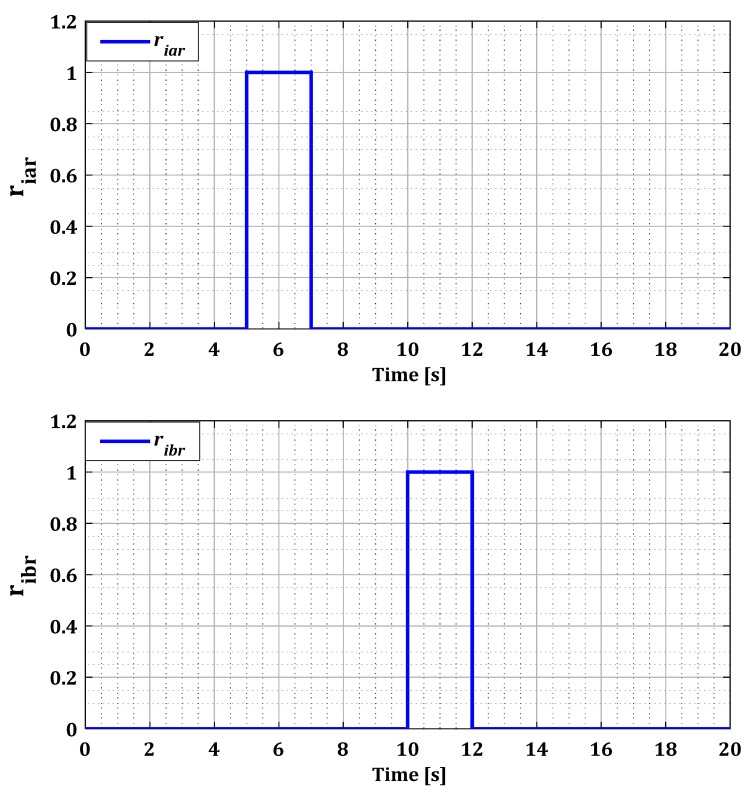

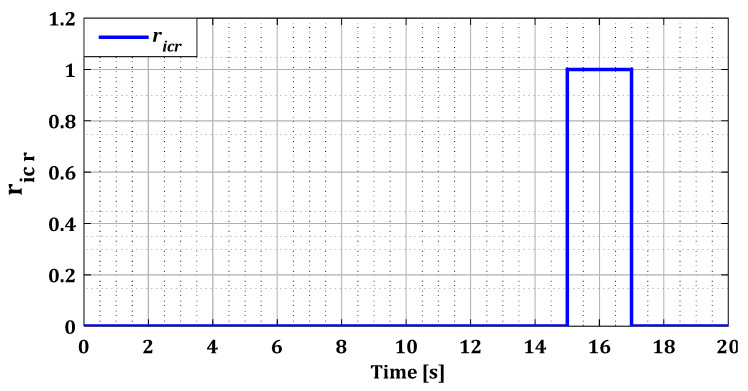
Residues $r_{iar}$, $r_{ibr}$, and $r_{icr}$ for single faults after the decision.

**Figure 13:**
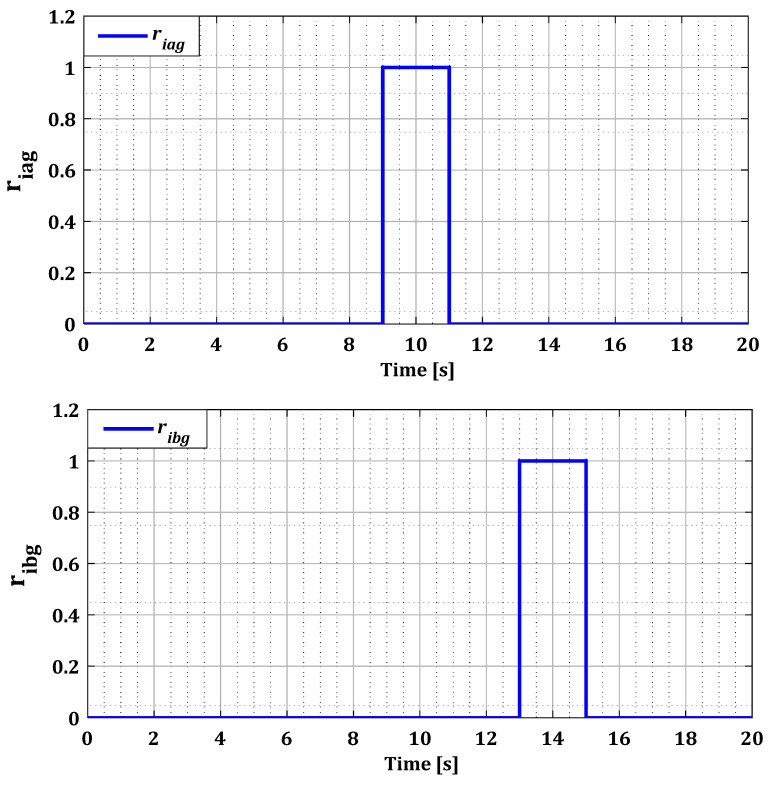

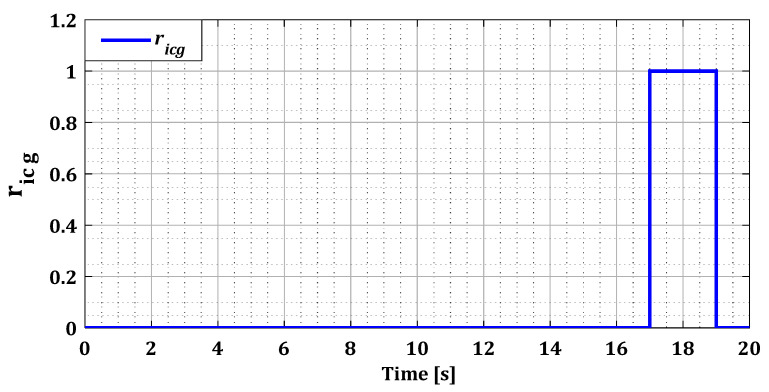
Residues $r_{iag}$, $r_{ibg}$, and $r_{icg}$ for single faults after the decision.

The decision results, represented in Figure 12 and Figure 13, highlight the faulty sensors during each fault period precisely indicate the duration of the appearance and disappearance of a sensor fault. The results we obtained demonstrate the effectiveness of our system in the accurate detection and localization of single faults in the current sensors in both converters.

### 4.2. Multiple Faults Scenario

**Figure 14:**
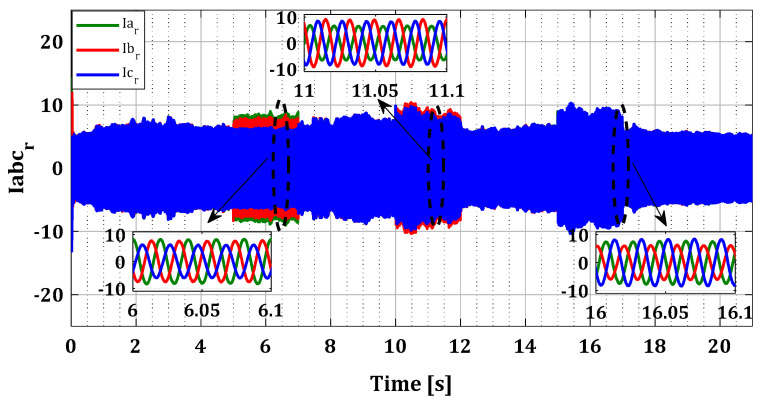
Rotor currents $I_{ar}$, $I_{br}$, and $I_{cr}$ for multiple faults.

**Figure 15:**
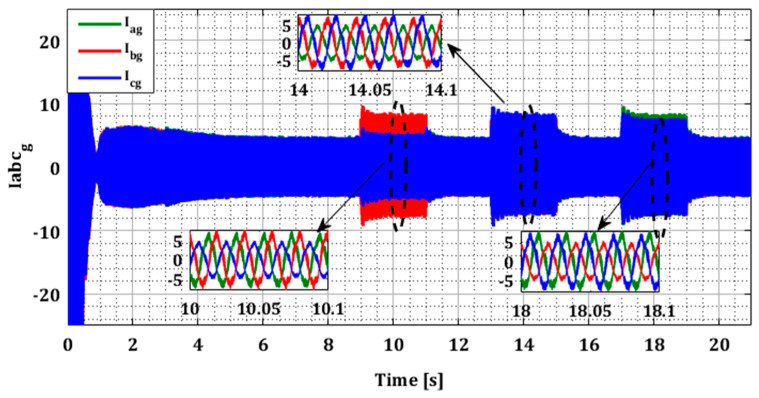
Rotor currents $I_{ag}$, $I_{bg}$, and $I_{cg}$ for multiple faults.

To demonstrate the effectiveness of our approach in the case of multiple faults in current sensors, we generated several simultaneous faults in the sensors of both converters, organized according to Table 1. Figure 14 and Figure 15 illustrate the current profiles for both converters, indicating the increase in current in each phase during the sensor failure period.

**Figure 16:**
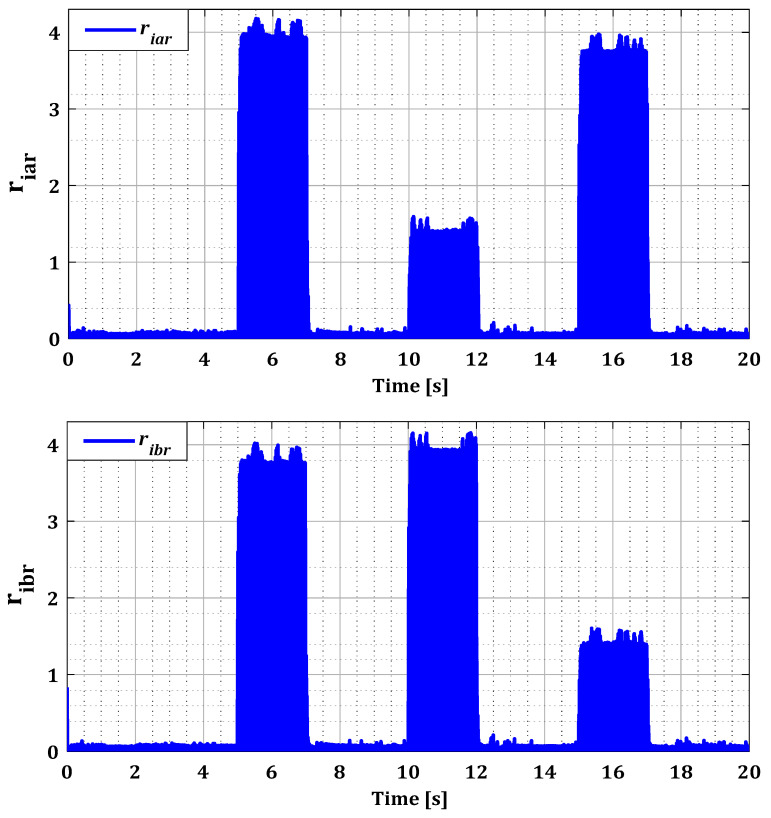

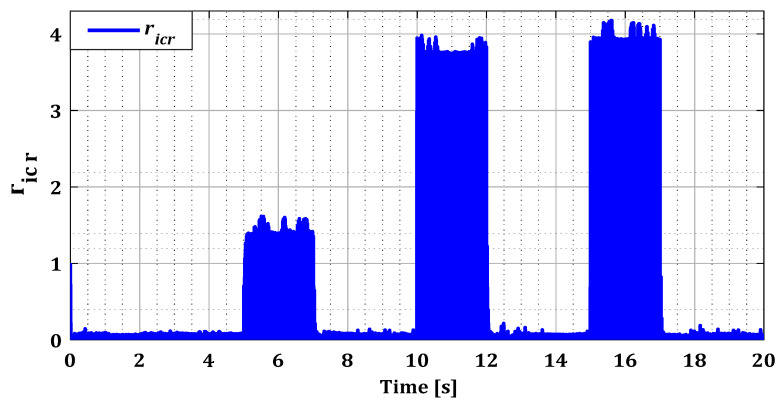
Residues $r_{iar}$, $r_{ibr}$, and $r_{icr}$ for multiple faults.

**Figure 17:**
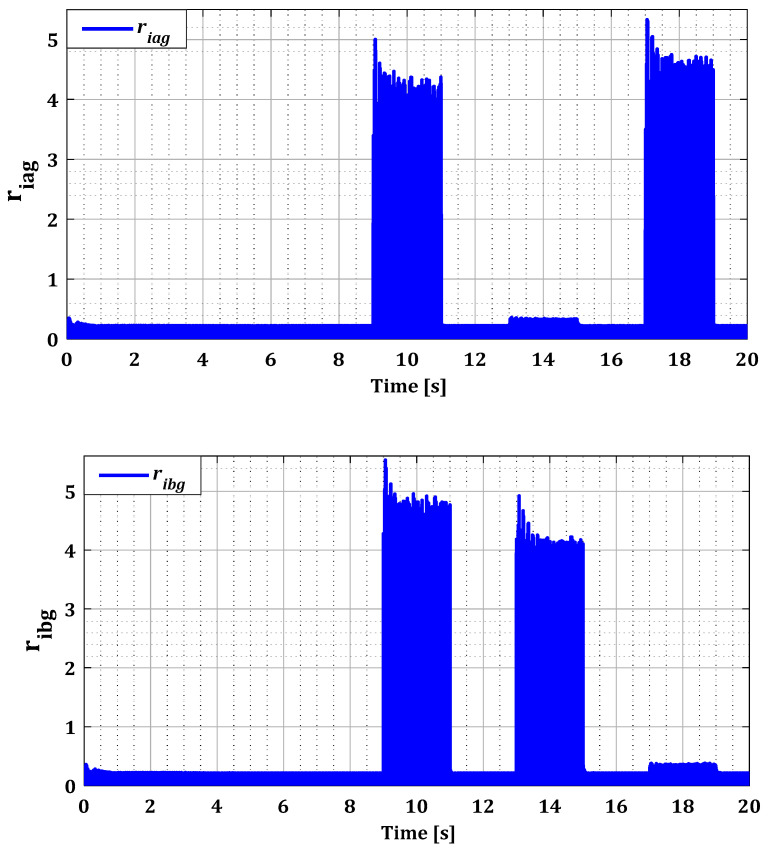

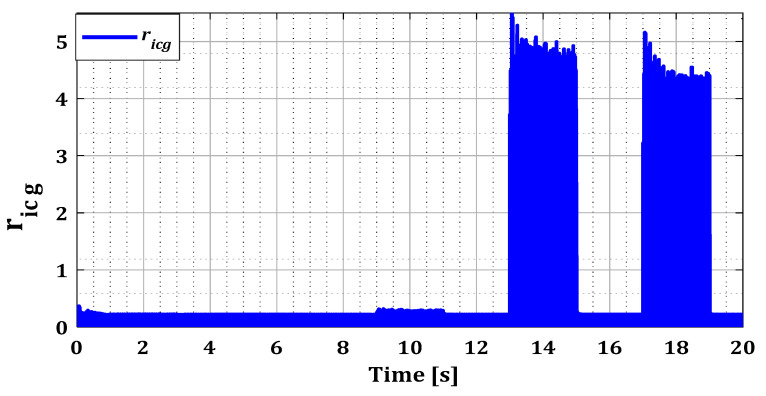
Residues $r_{iag}$, $r_{ibg}$, and $r_{icg}$ for multiple faults.

The results in Figure 16 and Figure 17 show that the value of each pair of residuals during the period of multiple faults is greater due to the presence of these faults. However, the third residual is lower compared to the others, indicating that two sensors are faulty, while the third one is in a normal state. For example, in the scenario of sensors failure (a) and (b) during period T12, illustrated in Figure 16, the residuals $r_{iar}$ and $r_{ibr}$ are higher than the residual $r_{icr}$, thus proving that there is a fault in both sensors of phases (a) and (b).

**Figure 18:**
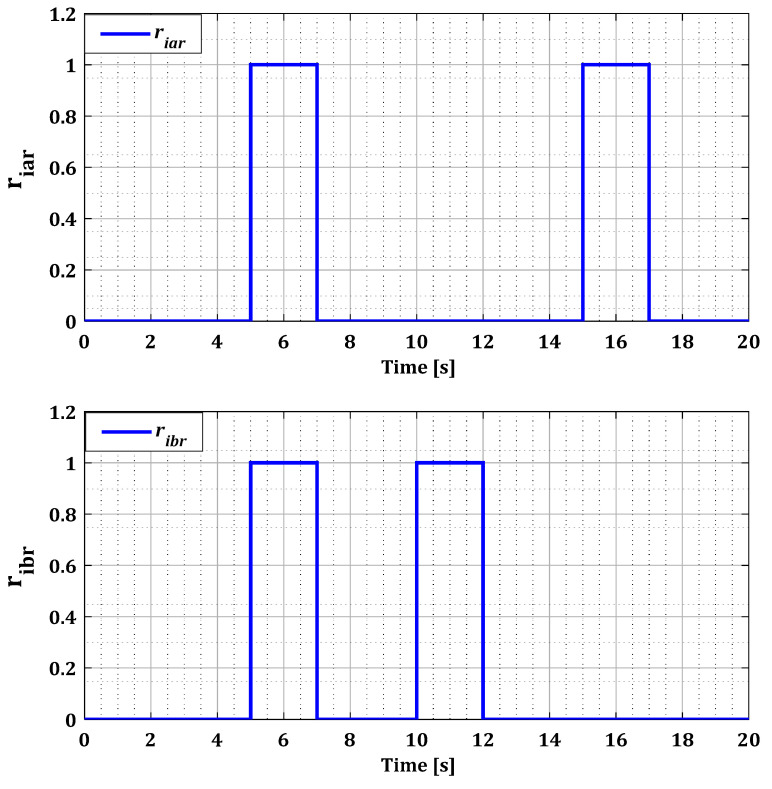

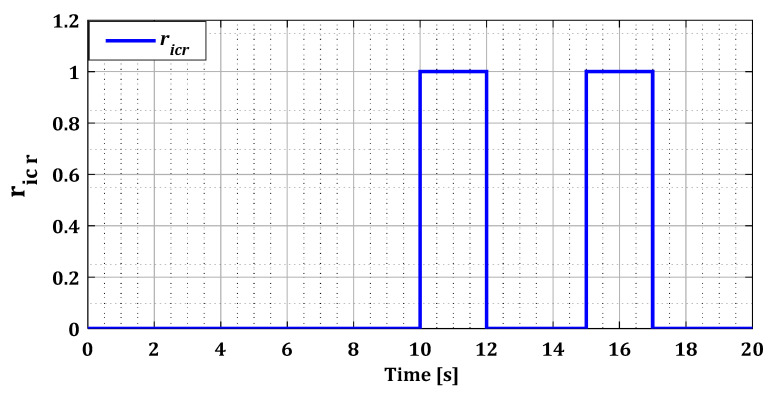
Residues $r_{iar}$, $r_{ibr}$, and $r_{icr}$ for multiple faults after the decision.

**Figure 19:**
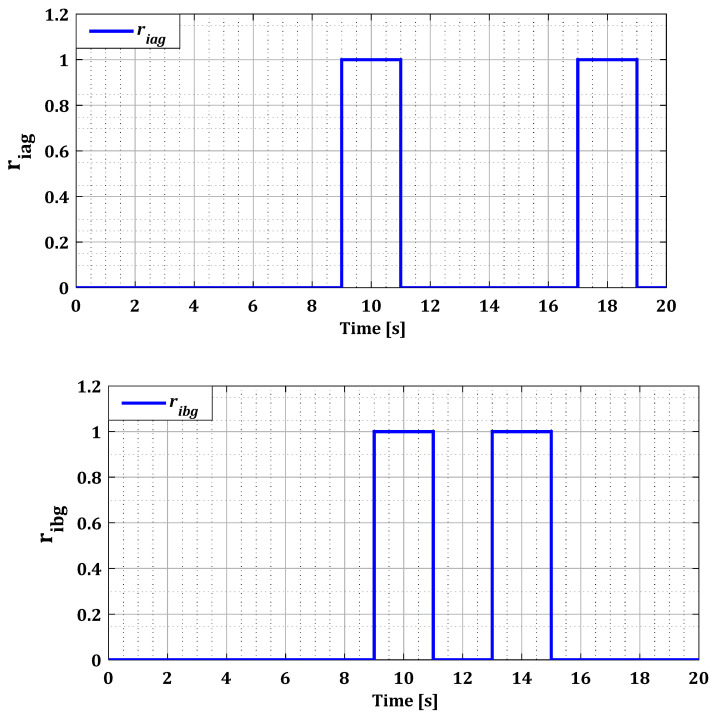

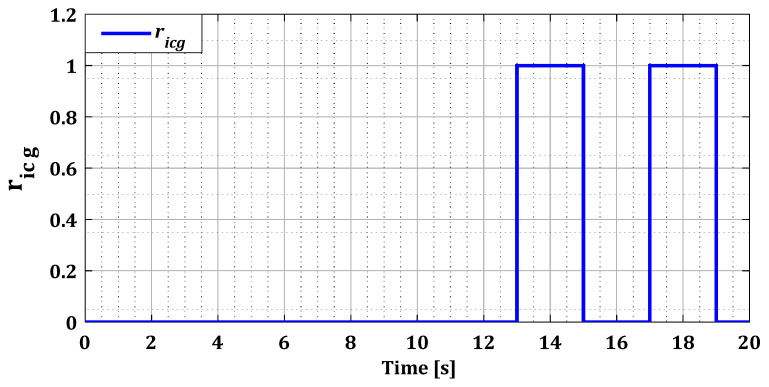
Residues $r_{iag}$, $r_{ibg}$, and $r_{icg}$ for multiple faults after the decision.

This behavior is replicated in all cases of simultaneous faults for both converters. These findings are confirmed by the decision block results presented in Figure 18 and Figure 19, which indicate accurately the duration of the appearance and disappearance of the sensor error. Based on the residuals found in all possible fault scenarios for both converters, it is noteworthy that these residuals quickly return to zero at the end of the fault period. This observation enhances the reliability and accuracy of our diagnostic approach when faults disappear.

The results obtained underline the effectiveness of our system in detecting and locating all potential multiple fault scenarios at the current sensors of both converters.

Table 2 provides a comparison of the diagnostic approach we have developed with other sensor fault diagnostic approaches.

## 5. Conclusions

This study provides a solution to the issue of diagnosing a wind turbine system based on a DFIG in the case of a failure of its current sensors, essential for the control of its converters (RSC, GSC). To design our diagnostic method, we developed two distinct mathematical models, each dedicated to a specific converter.

In the context of diagnostics, our approach relies on the application of the Kalman filter to the linear model on the grid side and the EKF using the Jacobian method to handle the nonlinearity of the DFIG. Both filters are implemented following the GOS. To generate fault residuals in the αβ frame, we then process them in a localization and isolation phase based on logical combination, followed by a statistical method for a better decision.

The test results demonstrate the effectiveness of our approach in precisely identifying all possible cases of single and multiple faults in the sensors of both converters at the abc real reference frame. This diagnostic approach demonstrates the advantage of our suggested method over other approaches presented in [29,30,31,32], which identify sensor faults in the (αβ) or (*dq*) frames. In addition, the contribution of this scientific paper lies in the diagnosis of current sensor faults on the GSC, compared with previous methods [29,30,31,32].

We can conclude that the obtained results open up numerous perspectives for furthering and extending this approach in the field of diagnosing faults in speed sensors, as well as electrical and mechanical faults within the system.

## Figures and Tables

**Table 1:** Test organization.

Type of Faults	Sensor Failure in Each Phase	Fault Period in RSC	Fault Period in GSC
	Phase (a)	T1 = [5; 7]	T1 = [9; 11]
Single fault	Phase (b)	T2 = [10; 12]	T2 = [13; 15]
	Phase (c)	T3 = [15; 17]	T3 = [17; 19]
	Phases (a), (b)	T12 = [5; 7]	T12 = [9; 11]
Multiple faults	Phases (a), (c)	T23 = [15; 17]	T23 = [17; 19]
	Phases (b), (c)	T32 = [10; 12]	T32 = [13; 15]

**Table 2:** Comparison of the diagnostic approach.

References	System Model	FDI Scheme	Linearization Method	Results
[13]	DFIG model in *dq*	Sliding mode observer	No linearization	Localizing faults in rotor current within the *αβ* or *dq* reference frames These methods are insufficient to accurately identify true single and multiple faults in rotor current sensors
[15]	DFIG model in *dq*	Bank of Luenberger observers	Transform the DFIG model into a linear parameter varying (LPV) form	
[17]	DFIG model in *αβ*	Luenberger observer	No linearization (steady speed)	
[18]	DFIG model in *dq*	Luenberger multiple observers (DOS)	Convert the DFIG model into a TS-type multimodel	
[21]	DFIG model in *αβ*	Bank of extended Kalman filters (DOS)	Linearization by Jacobian matrix	
[22]	DFIG model in *αβ*	Bank of Kalman filters (DOS)	Transform the DFIG model into a (LPV) form	
Current work	DFIG model in *αβ* GSC connection model in *αβ*	Bank of EKF (GOS) for DFIG Bank of Kalman filters linear (GOS) for GSC	Linearization by Jacobian matrix	Identify faulty sensors in each phase (a, b, c) for all possible scenarios of single or multiple additive faults for both converters

## Data Availability

Data are contained within the article.
